# Interpretation of serum magnesium concentrations in the presence of the systemic inflammatory response: a large patient cohort study

**DOI:** 10.1038/s41598-026-53085-3

**Published:** 2026-05-29

**Authors:** Donogh Maguire, Neil R. Syme, John Wadsworth, Dinesh Talwar, Donald C. McMillan

**Affiliations:** 1 Emergency Medicine Department, Glasgow Royal infirmary, 84 Castle Street, Glasgow, G4 0SF UK; 2 Academic Unit of Surgery, School of Medicine, University of Glasgow, New Lister Building, Royal Infirmary, Glasgow, G31 2ER UK; 3 Clinical Biochemistry, University Hospital Monklands, Airdrie, Lanarkshire ML6 0JS UK; 4 The Scottish Trace Element and Micronutrient Diagnostic Reference Laboratory, Department of Biochemistry, Royal Infirmary, Glasgow, G31 2ER UK

**Keywords:** Biochemistry, Biomarkers, Diseases, Medical research

## Abstract

The present study aimed to quantify the relationship between the systemic inflammatory response (SIR), measured by C-reactive protein (CRP) and albumin, and serum magnesium concentrations. Retrospective interrogation of laboratory computer databases at 4 centres between 1st October 2018 to 31st March 2019 provided results from patients in whom serum CRP and albumin had been measured together with serum magnesium concentrations. Analyte results were categorized into groups according to CRP and albumin. Patients (n = 9,624) were grouped according to serum magnesium concentrations: within normal range (0.75–1.10 mmol/L) (n = 6,313) (65%), low (< 0.75 mmol/L) (n = 3,152) (33%), or high (> 1.10) (n = 159) (2%). In those patients with a serum magnesium concentration below the normal range and a normal CRP, median serum magnesium concentrations were 0.70 (0.65–0.72) mmol/L, 0.68 (0.60–0.71) mmol/L, and 0.67 (0.59–0.72) mmol/L in patients with serum albumin concentrations ≥ 35 g/L, 25- <35, and < 25 g/L respectively. This represents a 4.2% change in serum magnesium concentrations among patients within the low serum magnesium group between mild inflammation and severe inflammation categories (p = 0.086). In those patients with a normal serum albumin concentration who were within the low serum magnesium category, serum magnesium concentrations were 0.70 (0.65–0.72) mmol/L, 0.69 (0.63–0.72) mmol/L, and 0.68 (0.63–0.71) mmol/L for CRP concentrations < 10 mg/dL, 11–79 mg/dL and > 80 mg/dL respectively. This represents a 2.9% change in serum magnesium concentrations for patients with normal serum albumin concentration between mild – severe inflammation (p = 0.109). Across a large, real-world clinical population, serum magnesium concentrations demonstrated limited variation in relation to the magnitude of the systemic inflammatory response and serum albumin concentration. Routine albumin adjustment of serum magnesium measurements may offer limited additional value as any observed differences in the present study were small and unlikely to be meaningful in clinical practice.

## Introduction

Magnesium is the second most abundant intracellular cation, with approximately 99% of total body magnesium localized to the intracellular compartment in bone and soft tissue, and approximately 1% localized in the circulation^1^. Magnesium is essential for over 300 enzymes, particularly those involved in energy metabolism and DNA damage repair^1,2^. A low circulating magnesium concentration has been reported to be important in several non-communicable disease states such as cardiometabloic health^3–6^. As circulating serum magnesium is approximately 30% bound to proteins (mainly albumin) and circulating magnesium may be perturbed by the inflammatory response^1^, it has been proposed that magnesium may be adjusted for albumin in analogous manner to calcium^7^ to increase clinical reliability.

A raised white cell count has long been recognised as part of the systemic inflammatory response. However, compared with acute phase proteins it lacks sensitivity to magnitude of tissue injury, particularly during the early post-operative period^8,9^. It has recently been reported in a small prospective study that the systemic inflammatory response (SIR) is associated with decreased serum magnesium and albumin concentrations following surgery^1^. However, the extent of the effect of SIR on serum magnesium and albumin concentrations remains unquantified in a large population-based study. Therefore, the aim of the present study was to quantify the impact of the magnitude of systemic inflammation (as evidenced by CRP), on serum magnesium and albumin concentrations in a large cohort of patients.

## Patients and methods

The study was registered with the UK Integrated Research Application System (IRAS number 20/NRS/0015) and categorised as an audit, not requiring not requiring individual patient informed consent or Research Ethics Committee approval. As per National Health Service (NHS) Scotland guidance, all methods of data extraction and analysis of the anonymised data were approved by NHS Greater Glasgow and Clyde Health Board (R&D reference: GN20ME077P). All methods were carried out in accordance with the relevant guidelines, regulations and approval. The analysis was conducted with the intent of developing local guidelines to aid in the interpretation of serum magnesium measurements.

Details of all requests for serum magnesium concentrations were extracted from the laboratory information systems of 4 Glasgow hospitals for the period between 1 st October 2018 to 31 st March 2019. These requests were made from secondary inpatient, secondary outpatient, and primary care health care providers. Data were anonymised and assigned a unique study number. Where an individual had repeat measurements, only the first was included in the study.

Serum magnesium concentration results were matched to CRP and albumin results obtained on the same calendar day using electronic laboratory patient identifiers. Any serum magnesium requests that were not accompanied by a contemporaneous request for serum CRP and albumin concentration assay were not included in the study (Table 1).

Serum magnesium concentrations (Isocitrate dehydrogenase)), serum CRP concentration (latex immunoassay)), and serum albumin concentration (Bromocresol purple) were measured using an automated analyser (Architect, Abbot Diagnosis, Maidenhead, UK) in the routine biochemistry laboratories. All samples were analysed following acceptable Internal Quality Control performance and there were no concerns regarding External Quality Assessment performance during this time.

Serum magnesium concentrations were categorized into groups according to CRP and albumin (CRP *≤* 10 mg/L, CRP 11– <80 mg/L and CRP *≥* 80 mg/L; and albumin *≥* 35 g/L, albumin 25–34 mg/L, and albumin < 25 g/L) (Tables 2 and 3).

**Table 1:** Age, Sex, CRP, Albumin and magnesium adjusted for albumin relative to low, normal and high serum magnesium concentrations (*n* = 9,624).

	Serum Magnesium Concentration (mmol/L)				
	Normal (0.75–1.05 mmol/L) (*n* = 6313)	Low (< 0.75 mmol/L) (*n* = 3152)	Normal vs. Low *P*-value*	High (> 1.05 mmol/L) (*n* = 159)	Normal vs. High *P*-value *
Serum Mg Concentration (mmol/L)	0.83 (0.79–0.88)	0.67 (0.61–0.71)		1.14 (1.09–1.26)	
Age (years)	61 (46–74)	65 (52–75)	< 0.001	61 (45–73)	0.928
Sex (male/female)	3246/3067	1347/1805	< 0.001^	92/72	0.017^
CRP (mg/dL)	8 (2–36)	19 (5–66)	< 0.001	26 (5–120)	< 0.001
Albumin (g/L)	36 (32–40)	32 (27–36)	< 0.001	34 (26–39)	< 0.001
Serum Mg concentration corrected relative to serum Albumin concentration (mmol/L)	0.86 (0.82–0.91)	0.71 (0.65–0.75)	< 0.001	1.18 (1.13–1.32)	< 0.001

*Mann Whitney U Test.

^Chi-squared analysis.

**Table 2:** Serum magnesium concentrations: (a) for patients within the normal limits of the normal reference interval (0.75–1.05 mmol/L) according to serum CRP and albumin concentrations (*n* = 6,313). (b) Adjusted for albumin for patients within the normal limits of the normal reference interval (0.75–1.05 mmol/L) according to serum CRP and albumin concentrations (*n* = 7,013).

(a)				
Normal serum magnesium (mmol/L) according to CRP and albumin categories	CRP *≤* 10 mg/L	CRP 11 – <80 mg/L	CRP *≥* 80 mg/L	Serum magnesium (mmol/L) according to albumin
Albumin *≥* 35 g/L	0.84 (0.80–0.88) (*n* = 2851)	0.84 (0.80–0.88) (*n* = 799)	0.85 (0.79–0.91) (*n* = 125)	0.84 (0.79–0.89) (*n* = 3775)
Albumin 34 - *≥*25 g/L	0.83 (0.79–0.87) (*n* = 574)	0.83 (0.78–0.88) (*n* = 963)	0.83 (0.79–0.89) (*n* = 496)	0.83 (0.79–0.88) (*n* = 2033)
Albumin < 25 g/L	0.83 (0.77–0.91) (*n* = 33)	0.82 (0.78–0.87) (*n* = 143)	0.84 (0.79–0.89) (*n* = 249)	0.83 (0.78–0.89) (*n* = 425)
Serum magnesium (mmol/L) according to CRP	0.83 (0.79–0.88) (*n* = 3485)	0.83 (0.79–0.88) (*n* = 1936)	0.83 (0.79–0.89) (*n* = 892)	

(b)				
	CRP *≤* 10 mg/L	CRP 11 – <80 mg/L	CRP *≥* 80 mg/L	
Serum magnesium adjusted for albumin (mmol/L) according to CRP	0.84 (0.80–0.89) (*n* = 3,673)	0.85 (0.80–0.91) (*n* = 2,251)	0.86 (0.81–0.93) (*n* = 1,089)	

**Table 3:** Serum magnesium concentrations (a) for patients below the lower limit of the normal reference interval (< 0.75 mmol/L) according to serum CRP and albumin concentrations (*n* = 3,152). (b) Adjusted for albumin for patients below the lower limit of the normal reference interval (< 0.75 mmol/L) according to serum CRP (*n* = 2,215).

(a)				
Serum magnesium (mmol/L) according to CRP and albumin categories	CRP *≤* 10 mg/L	CRP 11 – <80 mg/L	CRP *≥* 80 mg/L	Serum magnesium (mmol/L) according to albumin
Albumin *≥* 35 g/L	0.70 (0.65–0.72) (*n* = 735)	0.69 (0.63–0.72) (*n* = 307)	0.68 (0.63–0.71) (*n* = 61)	0.69 (0.64–0.72) (*n* = 1103)
Albumin 34 - *≥*25 g/L	0.68 (0.60–0.71) (*n* = 436)	0.67 (0.60–0.71) (*n* = 726)	0.66 (0.59–0.70) (*n* = 338)	0.67 (0.60–0.71) (*n* = 1500)
Albumin < 25 g/L	0.67 (0.59–0.72) (*n* = 32)	0.64 (0.56–0.70) (*n* = 182)	0.64 (0.57–0.70) (*n* = 275)	0.64 (0.57–0.70) (*n* = 490)
Serum magnesium (mmol/L) according to CRP	0.69 (0.63–0.72) (*n* = 1220)	0.67 (0.60–0.71) (*n* = 1247)	0.65 (0.59–0.70) (*n* = 685)	

(b)				
	CRP *≤* 10 mg/L	CRP 11 – <80 mg/L	CRP *≥* 80 mg/L	
Serum magnesium adjusted for albumin (mmol/L) according to CRP	0.69 (0.64–0.72) (*n* = 972)	0.68 (0.62–0.72) (*n* = 838)	0.68 (0.62–0.71) (*n* = 405)	

### Statistics

P values < 0.01 were considered statistically significant. Statistical analyses were performed using IBM SPSS, version 29 (Chicago, Ill). Data were presented as median and interquartile ranges (IQR). The cohorts were divided into groups according to CRP (*≤* 10, 11–80, > 80 mg/L) and albumin concentrations (< 25, 25–34, *≥* 35 g/L) as previously described^10^. The distribution of each parameter of magnesium status was compared across strata of CRP and albumin using the Kruskal-Wallis test, and between groups using Mann Whitney U Test. Categorical variables were analysed using Chi-squared test.

## Results

The proportions of patients included in the analysis from secondary inpatient, secondary outpatient and primary care were 80%, 18% and 2% respectively. From the study period, age, sex, CRP and/or albumin and serum magnesium concentrations data from 9,624 patients were included in the analysis. The majority of patients were > 60 years (55%), were female (51%) and had a serum magnesium in the normal range (66%).

Patients (*n* = 9,624) were grouped according to whether their serum magnesium concentrations were within the normal range (*n* = 6,313) (65%), were low (*n* = 3,152) (33%), or were high (*n* = 159) (2%). Comparison of patient characteristics with magnesium concentrations in the normal range with those with low and high magnesium concentrations are shown in Table 1. Patients with a low serum magnesium concentration were older (*p* < 0.001), female (*p* < 0.001), had higher CRP (*p* < 0.001), lower albumin (*p* < 0.001) and lower magnesium adjusted for albumin (*p* < 0.001) compared with those with magnesium concentrations in the normal range. Patients with high serum magnesium concentrations were similar in terms of age (*p* = 0.928) but had higher CRP (*p* < 0.001), lower albumin (*p* < 0.001) and higher serum magnesium concentration adjusted for albumin (*p* < 0.001) compared with those with serum magnesium concentrations in the normal range.

The relationship between CRP, albumin and magnesium in those patients with a serum magnesium concentration in the normal range are shown in Table 2. In those patients with a serum magnesium concentration in the normal range and a normal CRP, median serum magnesium concentrations were 0.84 (0.80–0.88) mmol/L, 0.83 (0.79–0.87) mmol/L, and 0.83 (0.77–0.91) mmol/L for serum albumin concentrations *≥* 35 g/L, 25- <35, and < 25 g/L respectively. This represents a 1.2% change in serum magnesium concentrations between normal and very low serum albumin concentrations for patients with CRP *≤* 10 mg/dL (*p* = 0.462). In those patients with a serum magnesium concentration in the normal range and a normal serum albumin concentration, median serum magnesium concentrations were 0.84 (0.80–0.88) mmol/L, 0.84 (0.80–0.88) mmol/L, and 0.85 (0.79–0.91) mmol/L for CRP concentrations *≤* 10 mg/dL, 11–79 mg/dL and *≥* 80 mg/dL respectively. This represents at 1.2% change in serum magnesium concentrations for patients with normal serum albumin concentration between mild – severe inflammation (*p* = 0.357).

The relationship between CRP, albumin and magnesium in those patients with a magnesium concentration below the normal range are shown in Table 3. In those patients with a serum magnesium concentration below the normal range and a normal CRP, median serum magnesium concentrations were 0.70 (0.65–0.72) mmol/L, 0.68 (0.60–0.71) mmol/L, and 0.67 (0.59–0.72) mmol/L in patients with serum albumin concentrations *≥* 35 g/L, 25- <35, and < 25 g/L respectively. This represents a 4.2% change in serum magnesium concentrations among patients within the low serum magnesium group between mild inflammation and severe inflammation categories (*p* = 0.086).

In those patients with a normal serum albumin concentration who were within the low serum magnesium category, serum magnesium concentrations were 0.70 (0.65–0.72) mmol/L, 0.69 (0.63–0.72) mmol/L, and 0.68 (0.63–0.71) mmol/L for CRP concentrations *≤* 10 mg/dL, 11–79 mg/dL and *≥* 80 mg/dL respectively. This represents a 2.9% change in serum magnesium concentrations for patients with normal serum albumin concentration between mild – severe inflammation (*p* = 0.109).

The relationship between CRP and albumin adjusted serum magnesium concentrations in those patients with an albumin adjusted serum magnesium concentration within the normal range and below the normal range are shown in Table 2(b) and 3(b) respectively. In those patients with albumin adjusted serum magnesium concentration below the normal range, median albumin adjusted serum magnesium concentrations were 0.69 (0.64–0.72), 0.68 (0.62–0.72), and 0.68 (0.62–0.71) in patients with CRP concentrations < 10 mg/dL, 11–79 mg/dL and *≥* 80 mg/dL respectively. This represents a 1.5% change in serum magnesium concentrations among patients within the low albumin adjusted serum magnesium group between mild inflammation and severe inflammation categories (*p* = 0.002).

## Discussion

The results of the present study show that low levels of serum magnesium are common in a large cohort of patients, and such low levels are associated with a greater inflammatory burden. Furthermore, the results show that elevated concentrations of serum magnesium, although not common, are also associated with a greater inflammatory burden. However, serum magnesium concentrations within the normal range were not associated with either CRP or albumin, and therefore the inflammatory burden per se does not appear to have a major influence on serum magnesium concentrations (certainly within the normal range). Therefore, although the relationship between serum magnesium and the inflammatory burden is apparent, the rationale for correcting such magnesium concentrations for albumin is weak.

The present manuscript addresses a question that has clinical relevance, specifically whether systemic inflammation (elevated C-reactive protein concentrations and hypoalbuminaemia) meaningfully influence serum magnesium concentrations, or more precisely their measurement, and whether albumin adjustment is warranted.

The basis of the present results is not clear. Although there is increasing evidence that the presence of a systemic inflammatory response confounds the interpretation of a number of serum micronutrients^11^, in the present study there was a relationship between both low and high magnesium concentrations and a greater inflammatory burden. One plausible explanation is that those patients with a high serum magnesium concentration represent a treated low magnesium group whereas those patients with a low magnesium represent an untreated group. Irrespective, it is clear that the undertreatment of magnesium concentrations is much more common than overtreatment.

Albumin is a negative acute phase protein commonly measured in clinical practice. Albumin is also quantitatively the most important circulating binding protein, and it is reported that between 25 and 35% of total serum magnesium is bound to it^12^. Kroll and Elin reported that serum albumin and magnesium concentrations were linearly related at high and low albumin concentrations; however, within the normal serum albumin reference interval, serum magnesium concentration remained independent of serum albumin concentration^12^. Therefore, the results of the present study are consistent with the latter study and question whether routine albumin adjustment of serum magnesium concentrations is justified.

Taken together the results of the present study are consistent with the concept that serum magnesium is a reliable measure in the context of SIR and may not require adjustment by albumin. It is not clear whether the absence of requirement for albumin adjustment also applies to other intracellular cations such as calcium^7^ and is worthy of further study.

## Limitations

Potential limitations of the present study are its observational nature, the lack of clinical data regarding indication for the investigation of magnesium (disease and supplementation) status in the included patients. However, given the large number of patients in the cohorts analysed in the present study, it would be unlikely that the associations identified were due to the sampling of a specific patient population. Moreover, given that routine clinical laboratory measures were used, the present observations can be readily externally validated.

## Conclusion

Across a large, real-world clinical population, serum magnesium concentrations demonstrated limited variation in relation to the magnitude of the systemic inflammatory response and serum albumin concentration. Routine albumin adjustment of serum magnesium measurements may offer limited additional value as any observed differences in the present study were small and unlikely to be meaningful in clinical practice.

## Data Availability

All data supporting the findings of this study are available within the paper and will be made available upon reasonable request to Professor Donogh Maguire.
